# A Multicenter Study on the Prognostic Value of Indicators Derived From Complete Blood Count in Glioblastoma

**DOI:** 10.1155/mi/5588098

**Published:** 2025-12-10

**Authors:** Shiqiang Hou, Qihong Gu, Tao Yang, Yiwen Hou, Min Wang, Yu Pan, Chunjing Jin, Ning Lin

**Affiliations:** ^1^ Department of Neurosurgery, The Affiliated Chuzhou Hospital of Anhui Medical University, The First People’s Hospital of Chuzhou, Chuzhou, 239000, China; ^2^ School of Public Health, Nantong University, Nantong, China, ntu.edu.cn; ^3^ Laboratory Medicine Center, The First Affiliated Hospital of Nanjing Medical University, Jiangsu Province Hospital, Nanjing, China, jsph.net; ^4^ Laboratory Medicine Center, The Affiliated Chuzhou Hospital of Anhui Medical University, The First People’s Hospital of Chuzhou, Chuzhou, 239000, China

**Keywords:** complete blood count, glioblastoma, indicator, prognosis, RDW

## Abstract

**Background:**

Previous studies have found that some indices derived from preoperative complete blood count (CBC) are closely related to the prognosis of glioma, but the results are inconsistent. This study comprehensively discussed the prognostic significance of the preoperative CBC index in patients with glioblastoma (GBM) through a multicenter study.

**Methods:**

In this multicenter study, we retrospectively analyzed clinical data from 143 GBM patients to evaluate the prognostic value of 12 preoperative CBC‐derived indicators: Neutrophil‐to‐lymphocyte ratio (NLR), platelet‐to‐lymphocyte ratio (PLR), monocyte‐to‐lymphocyte ratio (MLR), red cell distribution width (RDW), platelet distribution width (PDW), RDW‐to‐PDW (RPR), systemic inflammation index (SII), systemic inflammation response index (SIRI), hemoglobin‐to‐red cell distribution width ratio (HRR), platelet‐to‐basophil ratio (PBR), lymphocyte‐to‐basophil ratio (LBR), and eosinophil‐to‐lymphocyte ratio (ELR). Optimal cut‐off values for each indicator were determined using maximally selected rank statistics (MSRS). Survival outcomes were assessed by Kaplan–Meier analysis, and univariate and multivariate Cox regression were employed to identify independent prognostic factors. Furthermore, a nomogram was developed by integrating significant prognostic indicators to facilitate individualized prediction of survival in GBM patients.

**Results:**

The results showed that higher levels of NLR, PLR, MLR, RDW, PDW, and RPR were associated with shorter survival in GBM patients. In contrast, lower levels of ELR were associated with shorter survival in GBM patients. Among these, RDW (HR 1.905, 95% CI 1.114–3.258, *p* = 0.019), MLR (HR 1.603, 95% CI 1.029–2.496, *p* = 0.037), and ELR (HR 0.380, 95% CI 0.193–0.747, *p* = 0.005) emerged as an independent prognostic factors. The prognostic nomogram was constructed according to the three independent factors, which improved the accuracy of prognosis prediction (AUC = 0.702).

**Conclusion:**

Routine preoperative CBC parameters, particularly RDW, MLR, and ELR, serve as valuable complementary prognostic indicators for GBM patients. These accessible biomarkers warrant further validation through large‐sample, multicenter studies to solidify their clinical utility.

## 1. Introduction

Glioblastoma (GBM), a common primary brain tumor classified as WHO grade IV, predominantly arises in the frontal or temporal lobes, though it may develop throughout the central nervous system [1]. GBM is a highly malignant form of glioma classified as WHO grade IV, characterized by a rapid rate of cell division, vascular proliferation, and invasiveness [2]. Current therapeutic modalities for GBM consist of a combination of surgery (with the goal of maximal tumor resection), radiotherapy, and chemotherapy [3]. Despite therapeutic advances in the twenty‐first century, prognosis remains poor, with a median survival of approximately 15 months and a 5‐year survival rate below 5% [1, 4]. Given that nearly all patients experience disease recurrence, accurate assessment of prognosis, tumor burden, and risk stratification is essential for treatment planning. It is critical to find strong and trustworthy prognostic markers as soon as feasible to assist doctors in modifying the course of treatment to enhance the patient’s quality of life and extend their prognosis [5].

Existing research has highlighted limitations in current biomarker approaches for GBM. Many protein‐based biomarkers demonstrate poor detectability and reproducibility [6]. In addition, the few highly accurate genetic biomarkers have limited clinical application due to testing conditions and high costs [7]. In recent years, increasing attention has been focused on liquid biopsy—the development of noninvasive predictive biomarkers derived from hematological and serological parameters for various inflammatory diseases and malignancies [8–10]. Preoperative hematologic biomarkers are crucial indicators for cancer diagnosis and prognosis evaluation [11, 12]. Because they may partially represent the body’s tumor microenvironment [4, 13]. The preoperative complete blood count (CBC) test is mandatory for patients in almost all hospitals around the world and widely used as a common and simple test for cancer patients. Substantial evidence from numerous studies and meta‐analyses has established that multiple prognostic indicators derived from CBC are significantly associated with GBM outcomes [4, 14, 15].

Neutrophil‐to‐lymphocyte ratio (NLR), platelet‐to‐lymphocyte ratio (PLR), monocyte‐to‐lymphocyte ratio (MLR), systemic inflammation index (SII), and systemic inflammation response index (SIRI) are common immune‐inflammatory variables that may be useful as prognostic markers for a range of solid tumors [8, 16, 17]. Additionally, red cell distribution width (RDW) is also an important parameter of CBC. It is frequently used in clinical settings to identify the precise kind of anemia since it shows the variability of red blood cell size [18]. For a number of malignancies, increased RDW has been demonstrated to be a prognostic biomarker [19]. Similarly, platelet distribution width (PDW) is a direct parameter of platelet size variability in CBC. Inflammatory mediators can activate platelets, changing their shape from discoid to spherical and forming pseudopodia, which can raise PDW levels [12]. According to one research, PDW and RDW were both independent prognostic markers in GC, and the RDW‐to‐PDW (RPR) score may be used to accurately predict the prognosis of GC patients [20]. As for GBM, most of the published literature has similar findings. Clavreul et al. [4] discovered that preoperative NLR value was an independent risk factor for poor prognosis of GBM in multivariate analyses. Duan et al. [21] and Shi et al. [22] found that although high preoperative levels of NLR, MLR, PLR, and SII were prognostic risk factors for GBM patients, only SII was an independent risk factor.

Nevertheless, the prognostic significance of the aforementioned inflammatory indicators in GBM remains inconsistent across existing studies. Reports about these biomarkers in GBM are still relatively few, and the sample size is not enough. Thus, the goal of this research is to use multicenter studies to investigate and unearth the link between those indicators from CBC and GBM. Several new significant biomarkers derived from CBC are found in recent studies, including hemoglobin‐to‐red cell distribution width ratio (HRR), platelet‐to‐basophil ratio (PBR), lymphocyte‐to‐basophil ratio (LBR), and eosinophil‐to‐lymphocyte ratio (ELR). They have few reports in GBM, and their value is not well defined [23–25]. Accordingly, our study incorporates these novel markers as part of a systematic exploratory analysis.

## 2. Methods

### 2.1. Study Population

Retrospective clinical data from three medical centers between January 2017 and December 2021 made up the study population. The three medical centers were Jiangsu Province Hospital, Affiliated Hospital of Nantong University, and the First People’s Hospital of Chuzhou. Only patients who had surgical resection were included in this study to homogenize the patient cohort; patients who had a biopsy alone or no surgical intervention at all were excluded. Additionally, only patients with complete preoperative CBC information were included in further analyses. Following a cascade of censoring, 143 individuals with GBM were ultimately included in this research based on the following inclusion and exclusion criteria. The Institutional Review Board of the First People’s Hospital of Chuzhou approved this report and waived the need for obtaining informed consent. Ethical approval for this study was obtained and adhered to national and institutional research committee ethical standards and the Declaration of Helsinki.

### 2.2. Inclusion Criteria

(1) Patients with a primary diagnosis of GBM, that is, without radiation, chemotherapy, or resection, whose diagnosis was confirmed by pathologic evaluation. (2) Patients with completed routine pretreatment blood tests and adequate laboratory data. (3) Medical records provided baseline clinicopathologic information, such as age, sex, tumor size, and tumor grade, which was determined by immunohistochemistry staining. (4) The patient received routine radiation and chemotherapy after surgery. MGMT status was not mandatory for routine pathology reports at the centers, and IDH 1/2 mutation was not detected in the First People’s Hospital of Chuzhou before 2020. Therefore, the correlation with MGMT and IDH 1/2 mutation status was not analyzed in this study.

### 2.3. Exclusion Criteria

(1) individuals with comorbid conditions (hematologic illness, autoimmune disease, cardiovascular disease, and renal impairment). (2) A confirmed cancer. (3) Patients who do not have hyperpyrexia or overt infection. (4) Those who have a systemic or local infection. (5) Individuals using drugs for inflammatory conditions. (6) Absent follow‐up information.

### 2.4. Data Collection and Laboratory Measurements

Retrospective evaluation of the medical data of 792 patients undergoing curative resection for GBM between January 2017 and December 2021 was permitted by the institutional review board. As part of a standard preoperative workup, every patient had a full blood count taken 1–7 days after being admitted to the hospital. The CBC included measurement of neutrophils, lymphocytes, monocytes, platelets, hemoglobin (Hb), RDW, and PDW using the Sysmex XN‐1000 hematology analyzer (Sysmex Corp, Kobe, Japan). The calculation methods of these indicators derived from the CBC are described in Table 1.

**Table 1:** The calculation methods of the indicators derived from the CBC.

Name	Calculation methods	Name	Calculation methods
NLR	Neutrophils/lymphocytes	SII	Platelets × NLR
PLR	Platelets/lymphocytes	SIRI	Monocytes × NLR
MLR	Monocytes/lymphocytes	ELR	Eosinophils/lymphocytes
RPR	RDW/platelets	LBR	Lymphocytes/basophils
HRR	Hb/RDW	PBR	Platelets/basophils

**Figure 1:**
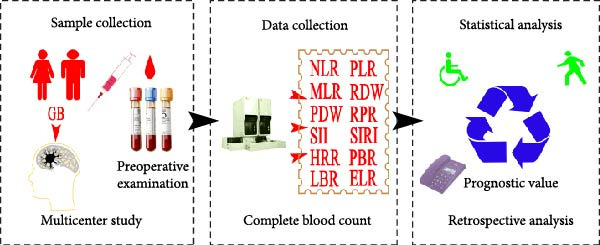
Scheme of this study.

Telephone interviews were used to check in with each patient on a regular basis. The most recent follow‐up was placed in December 2021. The date of the final follow‐up for survivors was used to determine the overall survival (OS), which was computed from the date of diagnosis to the date of death. The scheme of this study is shown in Figure 1.

### 2.5. Statistical Analysis

Categorical variables are shown as frequencies and percentages, whereas continuous variables are shown as medians. The data’s normality was evaluated using the Kolmogorov–Smirnov test. The chi‐squared test was used to assess categorical variables. The Spearman’s correlation coefficient was utilized to evaluate the connection between continuous variables. Implementing maximally selected rank statistics (MSRS) to identify the best cutoff thresholds for the hematological indices. Statistical significance of each identified cut‐off was rigorously assessed via permutation testing (1000 permutations, *p* < 0.05 required). Plotting Kaplan–Meier curves for patients with high and low hematological indicator values allowed for the analysis of survival differences between groups using the Wilcoxon test. By the log‐rank test in both univariate and multivariate Cox analyses, the predictive significance of hematological markers for survival was assessed. All variables that showed a potential association with OS in univariate analysis (*p* < 0.05) were initially considered for inclusion in the multivariate Cox proportional hazards model. Later, an enter selection procedure with a significance level of *p* < 0.05 was used to identify the most parsimonious set of independent prognostic factors. Sangerbox 3.0 is used to construct and test the nomogram, including Kaplan–Meier curve. All statistical analyses in this investigation were conducted using the Statistical Package for the Social Sciences (version 21 SPSS, Inc., Chicago, IL, USA) and R soft (R language 4.1.3 version, R Core Team, 2021), with *p* < 0.05 being deemed statistically significant.

## 3. Result

### 3.1. Patient Characteristics

The study comprised 143 GBM patients who had no systemic comorbidities or preoperative infectious illnesses and comprehensive preoperative hematologic markers. Table 2 summarizes the patients’ initial clinical and pathologic features. The maximal diameter of the contrast‐enhancing tumor was measured on axial slices of preoperative T1‐weighted gadolinium‐enhanced MRI. Tumor location was categorized by laterality (left or right hemisphere). The 143 research participants had a median age of 60 (9–84), of which 69 (48.25%) were under 60 and 74 (51.75%) were over 60. 65 (45.45%) of these patients were female, and 78 (54.55%) were male. The median values of preoperative NLR, PLR, MLR, RDW, PDW, RPR, SII, SIRI, HRR, PBR, LBR, and ELR were 2.64, 132.05, 0.27, 12.96, 15.60, 0.064, 528.21, 1.08, 10.55, 6700, 56, and 0.047, respectively.

**Table 2:** Clinical characteristics of 143 GBM patients.

Variables	Patients (range or %)	Variables	Patients (range or %)
Median age (year)	60.00 (9–84)	—	—
Gender	—	PDW	15.60 (9.30–22.10)
Male	78 (54.55)	<14.00	53 (37.10)
Female	65 (45.45)	≥14.00	90 (62.90)
Age (yr)	—	RPR	0.064 (0.41–0.184)
<60	69 (48.25)	<0.065	77 (53.80)
≥60	74 (51.75)	≥0.065	66 (46.20)
Tumor size (cm)	—	SII	528.21 (114.92–4022.16)
<3	58 (40.56)	<393.09	51 (35.70)
≥3	85 (59.44)	≥393.09	92 (64.30)
Tumor site	—	SIRI	1.08 (0.25–17.75)
Left	69 (48.25)	<1.89	110 (76.90)
Right	74 (51.75)	≥1.89	33 (23.10)
NLR	2.64 (0.62–17.72)	HRR	10.55 (4.60–14.19)
<2.35	59 (41.30)	<10.53	68 (47.60)
≥2.35	84 (58.70)	≥10.53	75 (52.40)
PLR	132.05 (27.38–474.47)	PBR	6700 (482–2,540,000)
<156.38	100 (69.90)	<5355	45 (31.50)
≥156.38	43 (30.10)	≥5355	98 (68.50)
MLR	0.27 (0.096–1.67)	LBR	56 (3.70–18,700)
<0.27	70 (49.00)	<33.58	26 (18.20)
≥0.27	73 (51.00)	≥33.58	117 (81.80)
RDW	12.96 (11.30–20.63)	ELR	0.047 (0.00–0.458)
<12.68	52 (36.40)	<0.01	21 (14.70)
≥12.68	91 (63.60)	≥0.01	122 (85.70)

### 3.2. Optimal Cutoff Values for the 12 Hematological Indicators

We implemented MSRS, the optimal method for survival data for cut‐off determination. As shown in Table 3, MSRS identified statistically significant cut‐offs (permutation *p* < 0.05) for three indicators, including NLR, PDW, and RDW. The optimal cutoff values were 1.801, 13.900, and 12.650, respectively.

**Table 3:** MSRS‐derived cutoff values for the 12 hematological indicators.

Variables	Maxstat (*M*)	*p*‐Value (condMC)	Optimal cutoff
NLR	3.0149	**0.045 ^∗^**	1.801
PLR	2.339	0.211	160.214
MLR	2.3342	0.214	0.2701149
RDW	3.2676	**0.012 ^∗^**	12.65
PDW	2.9254	**0.046 ^∗^**	13.9
RPR	2.4012	0.151	0.0804878
HRR	1.6805	0.624	8.985507
SIRI	1.6067	0.702	1.453053
SII	1.9349	0.441	488.7349
ELR	2.8936	0.056	0.1049383
LBR	1.9844	0.41	51
PBR	1.7395	0.568	5300

*Note:* Bold indicates statistical significance.

^∗^Represents *p* < 0.05.

### 3.3. Prognostic Role of Hematological Parameters on Kaplan–Meier Analysis

**Figure 2:**
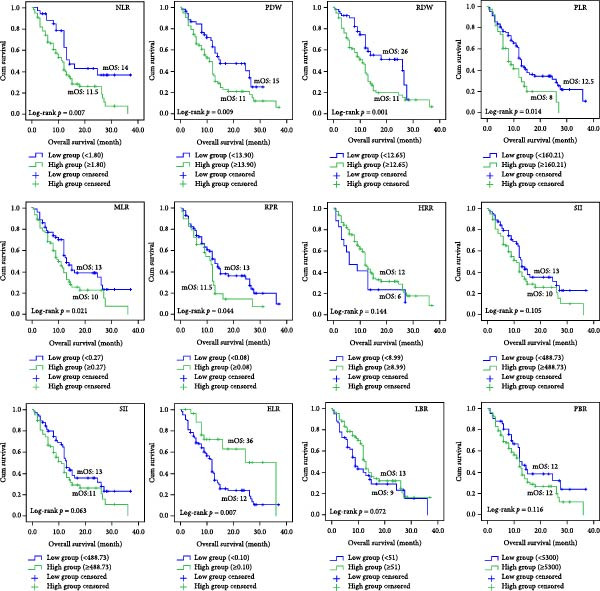
Kaplan–Meier survival analyses of 12 indicators based on the MSRS‐derived cut‐off values.

**Figure 3:**
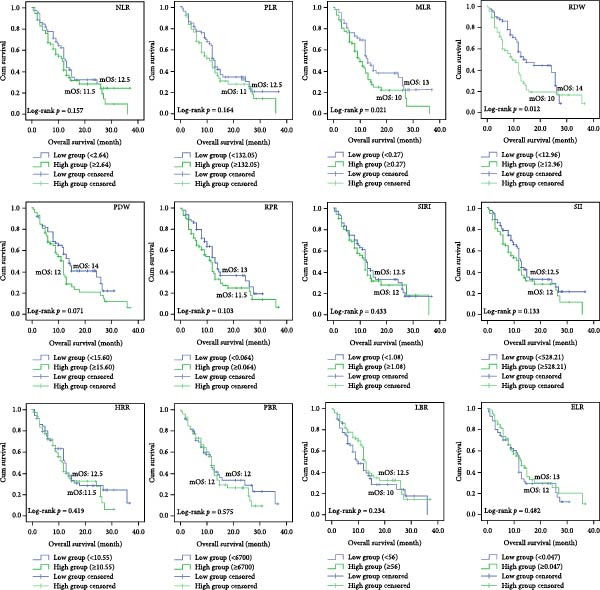
Kaplan–Meier survival analyses of 12 indicators based on the cohort median value.

We separated the 12 indicators obtained from CBC into two groups: high group and low group, based on the best cut‐off values by MSRS (Table 3). Then we performed the Kaplan–Meier survival analyses. According to the findings, the NLR high expression group’s median OS (mOS) time was 11.5 months, whereas the NLR low expression group’s mOS time was 14 months (*p* = 0.007). Patients in the low RDW group had an mOS of 26 months, while those in the high group with RDW ≥ 12.650 had a lower median OS of 11 months (*p* = 0.001). Patients in the low PDW group had a mOS of 15 months, whereas those in the high group with PDW > 13.900 had a poorer mOS of 11 months (*p* = 0.009, Figure 2). For the remaining nine markers without significant MSRS cut‐offs (Table 3), we found that the PLR high expression group’s mOS time was 8 months, whereas the PLR low expression group’s mOS time was 12.5 months (*p* = 0.014). The MLR high expression group’s mOS time was 10 months, whereas the MLR low expression group’s mOS time was 13 months (*p* = 0.021). The RPR high expression group’s mOS time was 11.5 months, whereas the RPR low expression group’s mOS time was 13 months (*p* = 0.044). The ELR high expression group’s mOS time was 36 months, whereas the ELR low expression group’s mOS time was 12 months (*p* = 0.007). Finally, we performed an additional sensitivity analysis using the cohort median value as an alternative threshold (Figure 3). Consistent with our primary findings, patients with RDW and MLR above the median continued to show significantly worse OS (*p* = 0.012 and *p* = 0.021, respectively).

### 3.4. Effects on Outcomes of Various Clinicopathological Factors and Hematological Indicators

**Figure 4:**
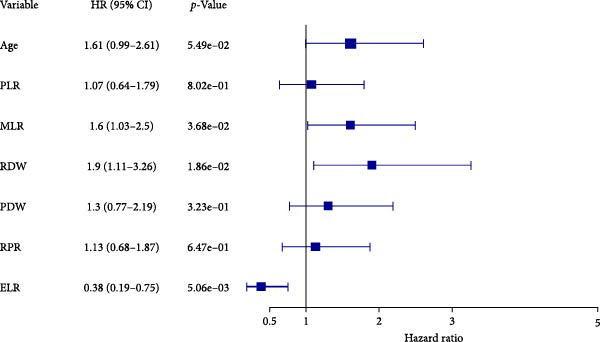
Forest plot of multifactor analysis.

The Cox proportional hazards models were employed to assess the factors that affect mortality. The univariate analysis revealed that the following were risk factors for a poor outcome: age > 60 years (HR 1.809, 95% CI 1.169–2.799; *p* = 0.008), NLR > 1.801 (HR 2.04, 95% CI 1.195–3.483, *p* = 0.009), PLR > 160.214 (HR 1.772, 95% CI 1.124–2.794, *p* = 0.014), MLR > 0.270 (HR 1.625, 95% CI 1.062–2.485, *p* = 0.025), RDW > 12.650 (HR 2.151, 95% CI 1.323–3.496, *p* = 0.002), PDW > 13.900 (HR 1.846, 95% CI 1.158–2.943, *p* = 0.010), RPR > 0.080 (HR 1.620, 95% CI 1.011–2.598, *p* = 0.045) and ELR > 0.105 (HR 0.411, 95% CI 0.212–0.796, *p* = 0.008 (Table 4). In the subsequent stage of multivariate analysis, we included components with *p*‐values less than 0.05 to prevent omitting clinically possible prognostic factors. Before multivariate analysis, we conducted variance inflation factor tests avoiding collinearity. The VIF of age, NLR, PLR, MLR, RDW, PDW, RPR, and ELR were 1.029, 5.127, 2.810, 3.437, 1.226, 1.277, 1.577, and 1.167, respectively. Hence, we exclude NLR (VIF > 5) from the final multivariate model. Age and preoperative PLR, MLR, RPR, and PDW xlost independent significance in the final multivariate model. The final results showed that RDW (HR 1.905, 95% CI 1.114–3.258, *p* = 0.019), MLR (HR 1.603, 95% CI 1.029–2.496, *p* = 0.037), and ELR (HR 0.380, 95% CI 0.193–0.747, *p* = 0.005) were independent risk factors, which is noteworthy that ELR was a protective factor (Table 4 and Figure 4).

**Table 4:** Univariate and multivariate Cox proportional hazards analyses of overall survival.

Variables	Univariate analysis			Multivariate analysis		
	HR	95% CI of HR	*p*‐Value	HR	95% CI of HR	*p*‐Value
Age > 60 years	1.809	1.169–2.799	**0.008 ^∗^**	1.608	0.990–2.613	0.055
Gender	0.767	0.503–1.169	0.218	—	—	—
Tumor site	0.887	0.581–1.353	0.577	—	—	—
Tumor diameter	1.074	0.699–1.648	0.746	—	—	—
NLR	2.04	1.195–3.483	**0.009 ^∗^**	—	—	—
PLR	1.772	1.124–2.794	**0.014 ^∗^**	1.068	0.636–1.795	0.802
MLR	1.625	1.062–2.485	**0.025 ^∗^**	1.603	1.029–2.496	**0.037 ^∗^**
RDW	2.151	1.323–3.496	**0.002 ^∗^**	1.905	1.114–3.258	**0.019 ^∗^**
PDW	1.846	1.158–2.943	**0.010 ^∗^**	1.301	0.772–2.190	0.323
RPR	1.620	1.011–2.598	**0.045 ^∗^**	1.126	0.677–1.875	0.647
SII	1.491	0.974–2.282	0.066	—	—	—
SIRI	1.432	0.923–2.221	0.109	—	—	—
HRR	0.655	0.368–1.164	0.149	—	—	—
PBR	1.459	0.904–2.353	0.122	—	—	—
LBR	0.684	0.447–1.046	0.080	—	—	—
ELR	0.411	0.212–0.796	**0.008 ^∗^**	0.380	0.193–0.747	**0.005 ^∗^**

*Note:* Bold indicates statistical significance.

^∗^Represents *p* < 0.05.

### 3.5. Predictive Nomogram for Prognosis in Patients With GBM

Based on three key prognostic factors—ELR, MLR, and RDW—a nomogram was developed to predict prognosis in GBM patients by integrating survival time, survival status, and these hematological parameters (Figure 5a). The nomogram demonstrated good discriminative ability, with a concordance index (C‐index) of 0.702. Kaplan–Meier survival analysis further validated the model, revealing significantly longer OS in the low‐risk group compared to the high‐risk group (*p* < 0.05, Figure 5b).

Figure 5Construction of the nomogram. (a) Nomogram, (b) Kaplan–Meier curves.

**Figure:**
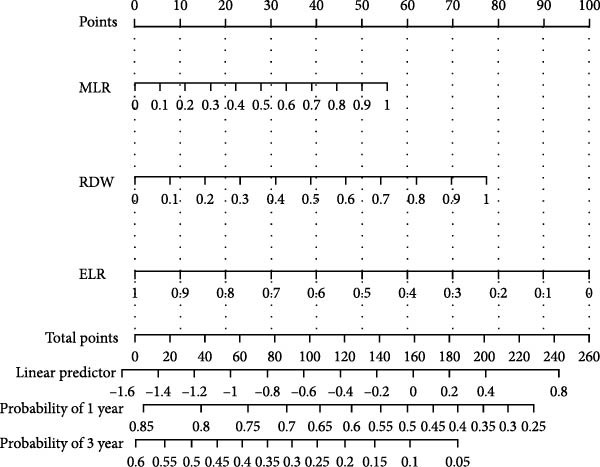
(a)

**Figure:**
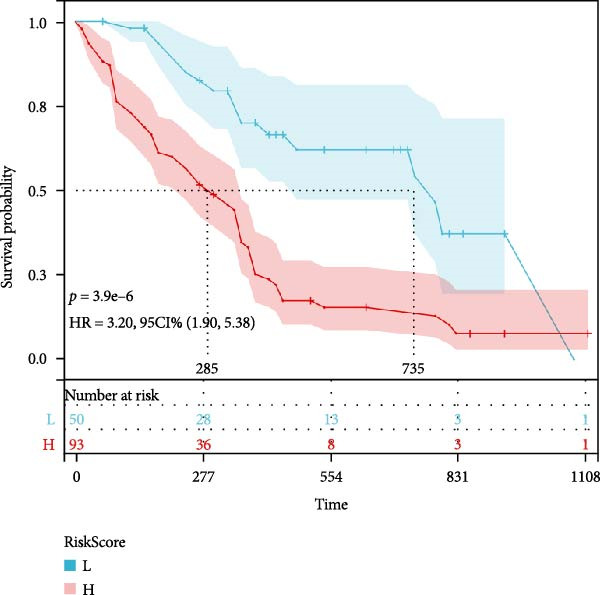
(b)

## 4. Discussion

RDW, MLR, and ELR were the independent prognosticators of GBM among the 12 inflammatory markers included in this investigation, while age, NLR, PLR, PDW, and RPR significantly negatively impacted patients’ OS in the Kaplan–Meier or univariate Cox regression models. Furthermore, a prognostic nomogram was developed by integrating the three identified independent factors: MLR, RDW, and ELR. Despite the stringent inclusion criteria which limited the study population to GBM patients only and precluded the incorporation of broader established risk factors (e.g., WHO grade I–IV), the nomogram maintained good discriminative ability, achieving a C‐index of 0.702.

In recent years, the immune‐inflammatory response in cellular carcinogenesis and tumor progression has been gradually investigated [26]. The host inflammatory response can induce the cellular microenvironment to produce inflammatory factors, thereby promoting tumorigenesis [27]. The tumor microenvironment can also produce inflammatory factors that regulate angiogenesis, apoptosis, and cell injury [18]. Therefore, the causal relationship between cancer and inflammation has attracted the attention of many scientists, and studies focusing on novel cancer‐associated inflammatory indicators are increasing [12]. Traditional methods for detecting inflammatory factors have some shortcomings, such as high testing costs, subjective analysis, unstable results, and are not suitable for screening high‐risk groups. As a routine test in all hospitals, CBC is a simple and inexpensive test that provides rapid detection of parameters relating to white blood cells, red blood cells, and platelets. As a sensitive reflection of immunohematologic changes, it has important clinical applications [28]. However, absolute single cell counts have poor specificity and are often influenced by various internal body environments. Therefore, most of the indicators included in this experiment are ratios of important parameters in the CBC, thus responding more accurately and comprehensively to the inflammatory response of the organism.

In our study, almost all CBC metrics that are closely associated with the diagnosis or prognosis of glioma or GBM in the existing literature were covered. NLR, PLR, and MLR, as the most reported metrics, they are valuable in the prognosis of various cancers, including glioma, especially NLR [11, 29]. According to a meta‐analysis, almost 30% of the studies have demonstrated a strong correlation between the NLR and cancer patients’ OS [30]. A robust connection was observed by Weng et al. [31] between glioma grade and preoperative NLR. Consistent with these studies, our previous study found that NLR was an independent predictor of OS in glioma patients compared with PLR and MLR [11]. As CBC‐derived markers of inflammation, SII and SIRI are often thought to be highly correlated with all‐cause and cardiovascular mortality [32, 33]. While both SIRI and NLR were reported to be independent prognostic markers in GBM, Model SIRI had better predictive power than Model NLR [34]. In glioma, there were inconsistent conclusions on RDW studies. RDW > 14.10% was reported by Liang et al. [35] to be a poor prognostic factor in 109 newly diagnosed GBM patients, and this assertion was subsequently supported by Kaisman‐Elbaz [36]. They found that normal Hb levels and RDW > 14% doubled postoperative survival in GBM patients (from 10.8 to 21.1 months) compared with normal Hb levels and RDW < 14%. Nevertheless, it has been argued that the immediate preoperative RDW value does not improve prognostic models in GBM by Kelly et al. [37]. These discrepancies may be due to differences in the method of determining thresholds, sample sizes, and inclusion of populations.

In addition, relatively few novel inflammatory markers RPR, PBR, LBR, ELR, and HRR have been reported in glioma or GBM. Schneider et al. [38] found that preoperative RPR can be utilized as an adjunctive prognostic predictor in GBM. According to Yang et al. [25], glioma patients with high PBR values had a worse prognosis. They also discovered that a forecast that combined PBR with a column–line graphic was accurate. Madhugiri et al. [23]. found that LBR was independently predictive of patient survival in GBM. ELR was discovered to be lower in high‐grade glioma patients than in low‐grade glioma patients by Huang et al. [24]. HRR, although not yet reported in glioma, has good predictive value in solid tumors, such as gastric [17], nasopharyngeal [39], and lung cancers [40]. Because the reported prognostic value of these important or novel inflammatory indicators in glioma or GBM varies widely, some are mutually supportive, and some are contradictory [41]. Therefore, we also included these indicators as exploratory factors in our multicenter study to further observe the value of these inflammatory indicators derived from CBC in the prognosis of GBM. Our primary analysis confirmed the prognostic value of novel ratios ELR as an independent protective factor for GBM. It may suggest that these specific cell types play a role in the GBM microenvironment. Nonetheless, as one of the first studies to report on these ratios in GBM, these findings provide a valuable reference for the field and highlight the need for further investigation in larger, independent cohorts.

Our research identifies a straightforward and trustworthy technique with clinical translational utility for predicting the prognosis of GBM patients. However, our study has some shortcomings. First, the main limitation of this study is the retrospective design, so we can’t completely exclude other situations that may affect the results, although we were very strict in the selection of patients. Second, due to the incidence of malignant brain tumors and strict inclusion and exclusion criteria, the number of patients included is not sufficient. Third, the most significant limitation of our study is the lack of comprehensive, uniformly tested MGMT promoter methylation and IDH mutation status data. These are cornerstone prognostic factors in GBM. Our inability to fully adjust for them in the primary multivariate model weakens the claim of the indicator’s complete “independence.” However, the biological rationale for the indicators reflecting systemic host factors (inflammation, nutrition) distinct from tumor‐intrinsic molecular pathways (MGMT/IDH), combined with extreme accessibility and low cost, suggests they could still offer valuable complementary or practical prognostic information, especially where molecular testing is unavailable. Future studies must prioritize concurrent evaluation of the CBC‐derived indicators and molecular markers. Prospective, multicenter, large‐scale tests are valuable for future monitoring and for in‐depth research on its molecular underpinnings. Of course, the mechanism of action between these factors and GBM also needs to be explored in depth.

## 5. Conclusion

12 potentially useful indicators obtained from CBC were examined in this study, and the findings demonstrated that RDW, MLR, and ELR are independent prognostic factors for GBM patients. The CBC test offers advantages of high reproducibility, widespread availability, and low cost. Consequently, it merits further investigation as it may serve, as a significant indicators of the dynamic status of GBM patients and support physicians in their decision‐making and therapy management.

**Nomenclature**
AUC: Area under the curve
ELR: Eosinophil‐to‐lymphocyte ratio
HRR: Hemoglobin‐to‐red cell distribution width
LBR: Lymphocyte‐to‐basophil ratio
MLR: Monocyte‐to‐lymphocyte ratio
NLR: Neutrophil‐to‐lymphocyte ratio
PDW: Platelet distribution width
PBR: Platelet‐to‐basophil ratio
PLR: Platelet‐to‐lymphocyte ratio
RDW: Red cell distribution width
RPR: Red cell distribution width‐to‐platelet ratio
SII: Systemic inflammation index
SIRI: Systemic inflammation response index.

## Ethics Statement

This study was approved by the Ethics Committee of the First People’s Hospital of Chuzhou (No. 83220464).

## Consent

The authors have nothing to report

## Disclosure

All authors reviewed the manuscript.

## Conflicts of Interest

The authors declares no conflicts of interest.

## Author Contributions

Shiqiang Hou and Chunjing Jin wrote the main manuscript text. Shiqiang Hou, Tao Yang, and Min Wang prepared figures and Tables. Qihong Gu, Yiwen Hou and Yu Pan conducted R software analysis and statistical analysis. Chunjing Jin and Ning Lin provided the revision for important intellectual content. Ning Lin provided the conception. Shiqiang Hou and Qihong Gu contributed equally to this work.

## Funding

This work was supported by the Scientific Research Foundation of Education Department of Anhui Province of China (Grant 2024AH040093), the Health Research Program of Anhui (Grant AHWJ2023A30052), the Chuzhou Science and Technology Program (Grants 2024YF007 and 2023ZD032), and the Health Research Program of Chuzhou (Grant CZWJ2024A001).

## Data Availability

The authors confirm that the data supporting the findings of this study are available within the article.
